# Morin improves learning and memory in healthy adult mice

**DOI:** 10.1002/brb3.3444

**Published:** 2024-02-26

**Authors:** Hilda Martínez‐Coria, Norma Serrano‐García, Héctor E. López‐Valdés, Gabriela Sinaí López‐Chávez, José Rivera‐Alvarez, Ángeles Romero‐Hernández, Francisca Fernández Valverde, Marisol Orozco‐Ibarra, Mónica Adriana Torres‐Ramos

**Affiliations:** ^1^ Departamento de Fisiología, Facultad de Medicina Universidad Nacional Autónoma de México Ciudad de México México; ^2^ Laboratorio de Neurofisiología Instituto Nacional de Neurología y Neurocirugía Manuel Velasco Suárez Ciudad de México México; ^3^ Ciencia Traslacional, laboratorio 4. Centro de Investigación sobre el Envejecimiento del Centro de Investigación y de Estudios Avanzados; Dirección de investigación, Instituto Nacional de Neurología y Neurocirugía Manuel Velasco Suárez Facultad de Ciencias Universidad Nacional Autónoma de México Ciudad de México México; ^4^ Laboratorio de Patología Experimental Instituto Nacional de Neurología y Neurocirugía Manuel Velasco Suárez Ciudad de México México; ^5^ Departamento de Bioquímica Instituto Nacional de Cardiología Ignacio Chávez Ciudad de México México

**Keywords:** astrocytes, BDNF, cognitive‐enhancing, cortex, hippocampus, pro‐BDNF, PSD95

## Abstract

**Background:**

Morin is a flavonoid found in many edible fruits. The hippocampus and entorhinal cortex play crucial roles in memory formation and consolidation. This study aimed to characterize the effect of morin on recognition and space memory in healthy C57BL/6 adult mice and explore the underlying molecular mechanism.

**Methods:**

Morin was administered i.p. at 1, 2.5, and 5 mg/kg/24 h for 10 days. The Morris water maze (MWM), novel object recognition, novel context recognition, and tasks were conducted 1 day after the last administration. The mice's brains underwent histological characterization, and their protein expression was examined using immunohistochemistry and Western blot techniques.

**Results:**

In the MWM and novel object recognition tests, mice treated with 1 mg/kg of morin exhibited a significant recognition index increase compared to the control group. Besides, they demonstrated faster memory acquisition during MWM training. Additionally, the expression of pro‐brain‐derived neurotrophic factor (BDNF), BDNF, and postsynaptic density protein 95 proteins in the hippocampus of treated mice showed a significant increase. In the entorhinal cortex, only the pro‐BDNF increased. Morin‐treated mice exhibited a significant increase in the hippocampus's number and length of dendrites.

**Conclusion:**

This study shows that morin improves recognition memory and spatial memory in healthy adult mice.

## Graphical abstract

Morin improves recognition and spatial memory in healthy adult mice. We suggest that the molecular mechanism for these effects involves the increased production and BDNF secretion by astrocytes.

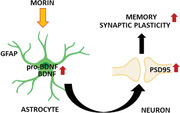

## 1 BACKGROUND

Some cognitive abilities, such as acquisition, storage, manipulation, and retrieval of information, usually decline with age and in some neurodegenerative diseases (Murman, 2015). Epidemiological and clinical evidence suggests that the level of education, an active lifestyle, and diet can improve cognitive function in healthy people and are protective against the rate of memory decline. Other research has established that learning and memory, as well as mood, can be strongly influenced by diet during development and in adulthood (Stangl & Thuret, 2009).

Polyphenols are among the largest groups of molecules present in diverse sources of plant origin, such as fruits and leaves. They are the most commonly found chemical compounds in food and herbal beverages consumed worldwide (Ebrahimi & Schluesener, 2012). Polyphenols are divided into several groups, and one of the most studied is flavonoids (Pandey & Rizvi, 2009). Flavonoids, in turn, can be subdivided, depending on their structure, into different subgroups, such as flavones, isoflavonoids, flavanones, flavanonols, flavanols or catechins, anthocyanins, chalcones, and flavonols (Nakanishi et al., 2020; Panche et al., 2016). Morin hydrate or morin (2‐(2,4‐dihydroxyphenyl)‐3,5,7‐trihydroxychromen‐4‐one; PubChem CID: 5281670) is a flavonol of small size (MW = 302.25) found in several fruits and vegetables, such as the white mulberry (*Morus alba* L.) (Lee et al., 2004), chestnuts (*Castanea sativa*, *Fagaceae* family) (Basile et al., 2000), almonds (*Prunus dulcis (Mill.) D. A. Webb*.) (Wijeratne et al., 2006), guava (*Psidium guajava* L.) (Pongsak Rattanachaikunsopon & Phumkhachorn, 2010), figs (*Ficus carica* L.), old fustic (*Chlorophora tinctoria*), and other members of the *Moraceae* family (Venkataraman, 1972). Morin is also present in beverages such as tea, coffee, red wine, and in some Chinese herbs (Caselli et al., 2016).

Experimental evidence from in vitro and in vivo studies indicates that morin exerts neuroprotective actions against several neuropathologies that show cognitive decline. For instance, in Alzheimer´s disease, in vitro studies show that morin inhibits the formation of amyloid and disaggregates amyloid fibers (Noor et al., 2012), inhibits β‐amyloid peptide (Aβ) fibrillogenesis (Kim et al., 2005), and its early stages of aggregation (Lemkul & Bevan, 2010). Moreover, morin blocks GSK3β‐induced tau phosphorylation (Gong et al., 2011). In Alzheimer's disease transgenic mouse models, morin shows different protection mechanisms; in APPswe/PS1dE9 mice, it restores cognitive functions, reduces the activated glial cells and Aβ production, and increases synaptic expression markers (Du et al., 2016). Moreover, morin attenuates tau hyperphosphorylation in the transgenic 3xTg‐AD mice model (Gong et al., 2011). In other neurodegenerative diseases, such as Parkinson's disease, in vitro studies showed that a decrease in the formation of reactive oxygen species and apoptosis in PC12 cells exposed to MPP+ (Zhang et al., 2010) as well as reduced lipid membrane damage by α‐synuclein aggregates (Caruana et al., 2012), and significantly increased motor abilities (Zhang et al., 2010). Furthermore, morin protects neurons and oligodendrocytes from excitotoxic cell death (Gottlieb et al., 2006; Ibarretxe et al., 2006) and significantly improves spatial memory in an animal stroke model (Gottlieb et al., 2006). Recently, morin was described to produce an antipsychotic‐like activity in mice, possibly mediated via mechanisms related to decreased inflammation and enhancement of GABAergic neurotransmission, brain‐derived neurotrophic factor (BDNF), and suppression of NADPH‐oxidase, which induce oxidative damage and neuroinflammation (Ben‐Azu et al., 2019; Ben‐Azu et al., 2018, 2018, 2018, 2018). Given the association between psychosocial stress and neuropsychiatric disorders, morin also has been tested in animal models of social stress where it showed to reduce inflammatory mediators, protect hippocampus from damage, and improve motor and memory impairments induced by psychosocial stress (Ben‐Azu et al., 2020; Elizabeth et al., 2020). Sleep deprivation is another stress factor that can cause several changes that induce learning and memory deficits (Chen et al., 2023), and morin treatment has shown significant reduction in memory impairment, anxiety‐like behavior, and neuronal damage in a mouse model of sleep deprivation (Olonode et al., 2019).

BDNF plays a crucial role in processes that underlie learning and memory, such as excitatory synaptic transmission, regulation of the structure and functions of different neuronal circuits throughout life, and activity‐dependent changes in synaptic strength and plasticity, among others, suggesting a pivotal role in these cognitive functions (Bekinschtein et al., 2008; Poo, 2001; Yamada & Nabeshima, 2004). Recently, an essential role in Hebbian‐type long‐term potentiation, long‐term depression (LTD), and homeostatic synaptic plasticity has been established for morin (Wang et al., 2012). In the adult brain, BDNF is synthesized and secreted by neurons and glial cells, such as microglia and astrocytes, and this neurotrophin regulates structural and physiological aspects of synapses ranging from short‐term to long‐lasting in many brain regions, including the hippocampus (Lu et al., 2014). BDNF is required for learning and memory processes, and its increase is also correlated to changes in the number, size, and shape of dendrites, which are part of the structural synaptic plasticity (Zagrebelsky et al., 2020). On the other hand, reduced BDNF levels have been reported in disorders that produce cognitive impairments, including chronic stress, aging, and Alzheimer's diseases, principally in memory‐related structures, like the hippocampus and entorhinal and frontal cortices (Brigadski & Leßmann, 2020; Miranda et al., 2019). Moreover, it is known that the downregulation of BDNF decreases learning and memory (Mizuno et al., 2003).

There has been a growing interest over the last decade in natural molecules with the potential to improve cognitive ability not only in sick people but also in healthy people, and several studies in humans and animals have shown that consuming flavonoids is associated with better cognitive function (Spencer, 2008). However, there is limited information regarding the effect of pure molecules as a cognitive enhancer, particularly in learning and memory.

Thus, the present study was designed to examine whether morin functions as a cognitive enhancer on the memory of healthy mice using the Morris water maze (MWM) and object recognition tasks with or without novel context and to determine some of the molecules related to such effects. The behavioral tests applied in this study are among the most widely used to assess spatial memory (MWM) and recognition memory (CNOR and NOR). MWM is a test commonly used to assess mainly hippocampal performance in spatial navigation and reference memory, whereas CNOR and NOR assess mainly perirhinal cortex performance in recognition memory (Antunes & Biala, 2012; Vorhees & Williams, 2006).

## 2 METHODS

### 2.1 Reagents

Morin was obtained from Sigma‐Aldrich Co. (M4008). Anti‐GFAP polyclonal was from DAKO (RRID:AB_10013382), anti‐BDNF (RRID:AB_10862052), and anti‐postsynaptic density protein 95 (PSD95) (RRID:AB_444362) were from Abcam. Anti‐pro‐BDNF monoclonal antibody (RRID:AB_1128219) and horseradish peroxidase‐conjugated goat anti‐mouse IgG (RRID:AB_631736) were obtained from Santa Cruz Biotechnology, Inc. Goat anti‐rabbit IgG H&L, Alexa Fluor 488 (RRID:AB_2576217), and rabbit anti‐mouse IgG H&L, Alexa Fluor 594 (RRID:AB_2534109), were used for fluorescence. All other reagents were of analytical grade and are commercially available.

### 2.2 Animals

C57BL/6 (RRID:MGI:2159769) male adult mice (3–4 months old, weighing between 25 and 35 g) were obtained from the vivarium at the Instituto Nacional de Neurología y Neurocirugía Manuel Velasco Suárez. The mice were kept in standard conditions (12/12 h light/dark cycle, 21 ± 2°C, and 40% relative humidity) with access to food and water ad libitum. The experiments were performed according to the Official Mexican Standard on Technical Specifications for the Production, Care and Use of Laboratory Animals (NOM‐062‐ZOO‐1999). The institutional ethical committee for the Care and Use of Laboratory Animals (CICUAL‐INNNMVS) evaluated and approved the protocol on March 29, 2021, approval number INNN‐138/19.

### 2.3 Experimental design

Morin was dissolved in DMSO and diluted with saline solution. Mice were divided into four groups: vehicle (DMSO in saline solution 0.25 mL/L/24 h i.p. for 10 days) and morin (1, 2.5, and 5 mg/kg/24 h i.p. for 10 days). Ten mice per group were used for each test of learning and memory, whereas 10 additional mice per group were used for molecular studies. As it is known that DMSO can cause deleterious effects or epigenetic changes at concentrations higher than 1% (V/V) and 0.1% (V/V), respectively, in this work we used a concentration much lower than those reported to cause such effects (de Abreu Costa et al., 2017; Verheijen et al., 2019).

### 2.4 Behavioral test

#### 2.4.1 Object recognition and object recognition in a novel context

Morin and vehicle‐treated mice were first tested on CNOR and Novel NOR, mainly hippocampal and cortex‐dependent. The following groups were tested for the two experiments: DMSO, morin 1, 2.5, and 5 mg/kg. The habituation for both tasks was performed on days 7–9 (of 10 days of treatment) by placing the mice in an empty arena for 5 min each day.

As previously described, 10 mice per group were trained in a NOR task (Martinez‐Coria et al., 2010). The following four groups were tested: DMSO, morin 1, 2.5, and 5 mg/kg. The animals were trained by letting them explore two identical objects at opposite ends of the arena for 5 min. The next day, mice were tested for 3 min with one familiar object and one novel object of similar dimensions. The recognition index (RI) represents the percentage of time mice spent exploring the novel object.

In order to study the recognition by a different experimental approach, another four groups (DMSO, morin 1, 2.5, and 5 mg/kg) were trained in a novel context task as follows: The animals were trained by letting them explore a pair of identical objects in a first arena (context A) for 5 min. After 90 min, the mice were placed in a second arena (context B) with another pair of identical objects and allowed to explore for 5 min. Finally, 24 h later, mice were tested for 3 min with one familiar object in context A and one object out of context from the second arena (B). The RI represents the percentage of time mice spent exploring the object out of context.

### 2.5 Morris water maze

Hippocampal‐dependent learning and memory were examined using the MWM following standard protocols (Martinez‐Coria et al., 2010). After 6 days of treatment, DMSO and morin (1 mg/kg) mice groups were trained in a 1.2‐meter diameter circular pool filled with opaque water at 21°C. During the 4 days of training, mice were placed into the pool and allowed to find a submerged escape platform (4 trials/day). On the 5th day, the platform was removed to assess memory retention of the former platform location. Latency to cross the area and the number of crosses were measured.

### 2.6 Tissue processing

Tissue processing for immunohistochemical detection of proteins and quantification was performed following previously reported methods (Serrano‐García et al., 2018).

The mice were sacrificed by pentobarbital overdose and cardiac perfusion with 0.01 M phosphate‐buffered saline. Their brains were removed and cut along the sagittal midline. The half brain was micro‐dissected into the hippocampus and entorhinal cortex and frozen on dry ice for subsequent biochemical analysis, whereas the other half was fixed in 4% paraformaldehyde (pH‐7.4, 48 h).

Fixed brains were embedded in paraffin and serially sectioned to obtain sagittal cuts of 7 µm to analyze the hippocampus. Then, GFAP and IL‐4 were determined by immunostaining. The stained slices were examined at 10× magnification; micrographs were used to determine the number of positive cells per slice for GFAP and integrated optical density for IL‐4. The quantitative analysis of the GFAP‐positive cells was carried out using ImageJ version 1.45. The GFAP‐immunoreactive cells were manually counted at 10× in CA1, CA2, CA3, CA4, molecular layer dorsal (ML dorsal), molecular layer ventral (ML ventral), granular layer dorsal (GL dorsal), granular layer ventral (GL ventral), mossy fibers, radiate layer, and oriens layer. Measurements were taken of four to six tissue sections from each animal in the entire layer of the hippocampus, and three animals were analyzed for each condition. Results were expressed as numbers of GFAP‐positive cells.

### 2.7 Western blot

Equal amounts of protein (20–50 µg, depending on the protein of interest) were separated on 10% or 4% to 12% Bis/Tris gels (Bio‐Rad) and transferred to a nitrocellulose membrane. Membranes were blocked for 1 h in 5% (v/v) suspension of nonfat milk Tris‐buffered saline (pH 7.5) and incubated overnight with primary antibody at 4°C. The primary antibodies used for Western blots are summarized above. After washing, the membranes were incubated with adjusted secondary antibodies coupled to horseradish peroxidase. Quantitative densiometric analyses were performed using ImageJ 1.4 software (NIH).

### 2.8 Golgi–Cox staining of neural dendrites

**FIGURE 1:**
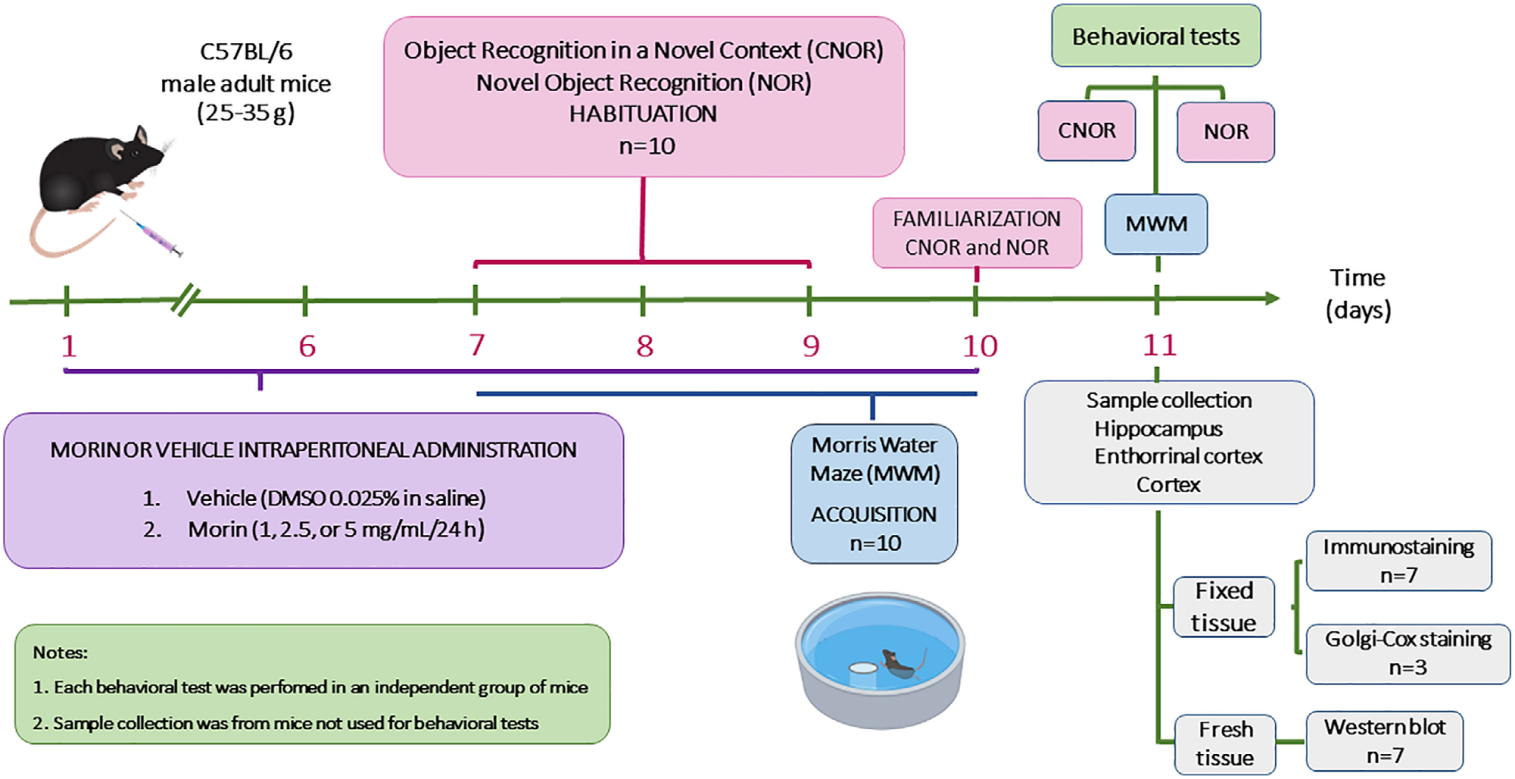
The figure shows the timeline of the experimental procedures. The experimental approach is represented on a timeline by a sequence of colored boxes paired with a colored line, which highlights pertinent details about the method and the procedure's time frame.

Coronal sections (Bregma 2.40 mm) of mouse brain (14 weeks old), control and treated with 1 mg/kg morin, were stained using the Golgi–Cox staining of neural dendritic silver impregnation technique. We used an FD Rapid GolgiStain Kit, FD NeuroTechnologies #PK401. The photographs were taken under a NIKON KCC‐REM‐NCY‐E200 DMV model Eclipse E200LED MV R microscope. Fifteen neurons per individual were chosen, and those neurons in which their individuality was evident were selected, and the dendrites were drawn in Paint 3D to facilitate visualization. The dendrites of each selected neuron were counted for their subsequent statistical analysis with a two‐way *t*‐test, considering *n* = 45 for each treatment. Finally, the dendritic length was evaluated with the ImageJ 1.4 software, and the images were converted to an 8‐bit format to transfer them to the NeuronStudio program and perform the Sholl analysis for each neuron. Subsequently, all the values of the dendrite length column were then added (values reflected in the results of the Sholl analysis). Figure 1 summarizes the experimental strategy used in this work.

### 2.9 Statistical analysis

Animals were randomly assigned to treatment versus control groups in this double‐blind study. For immunohistochemistry and western blotting, images were processed using ImageJ automated software and the values obtained were analyzed with Student's *t*‐test. The RI values between familiar and novel objects obtained during NOR and CNOR tests were compared with one‐way ANOVA (between groups), followed by multiple comparisons tests with statistical significance determined using Tukey's method. The MWM test was analyzed by repeated‐measures ANOVA and Turkey post hoc for between‐group interaction and one‐tailed unpaired *t*‐student for both trials of the spatial training retention probe. To analyze differences in dendrites, the Mann–Whitney *U* test was used. All values were analyzed with GraphPad Prism 9.0.0 and all graphs were shown as the mean ± standard error of the mean and a *p* value of < 0.05 was considered statistically significant.

## 3 RESULTS

### 3.1 Behavior: morin improves learning and memory

**FIGURE 2:**
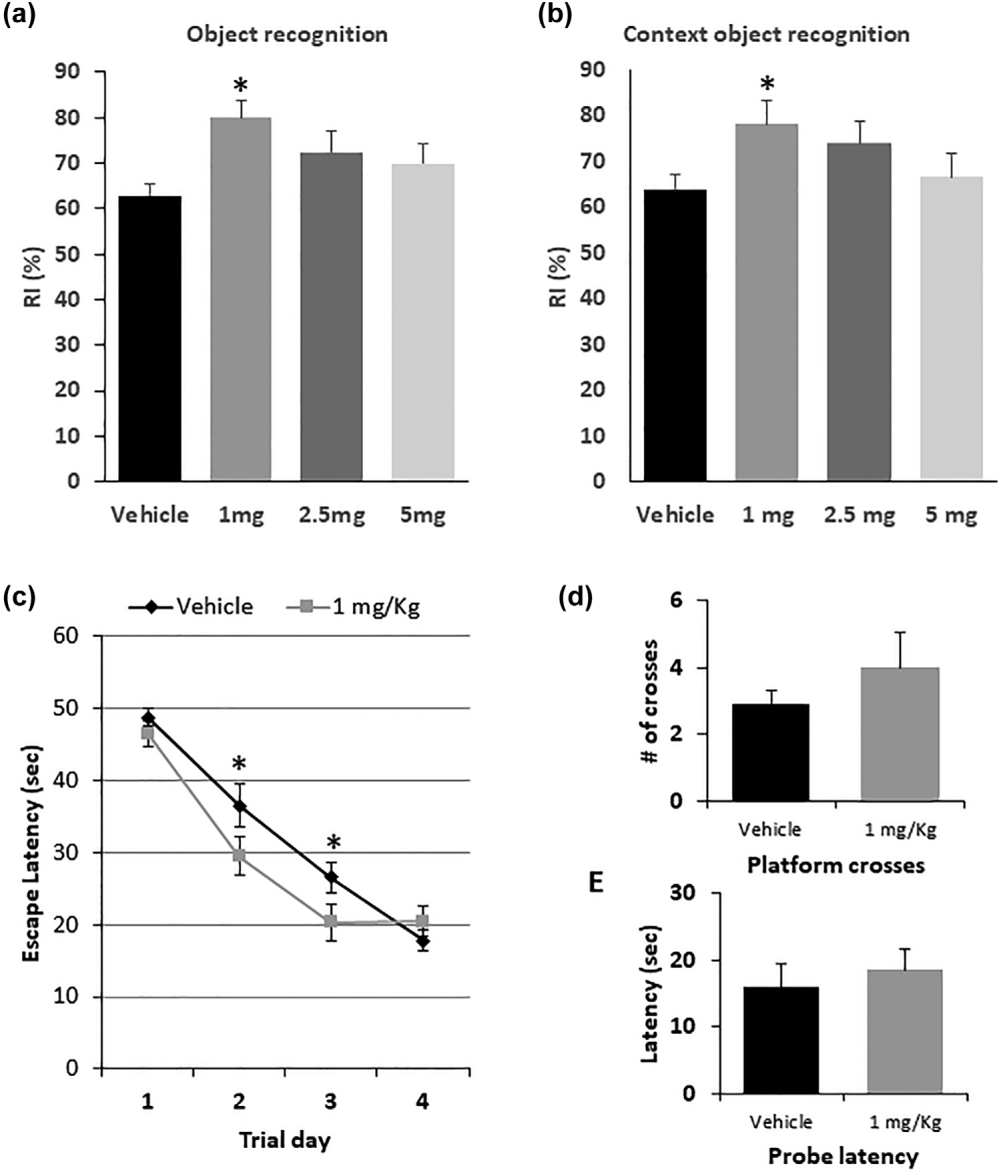
Morin effect on learning and memory. C57BL/6 mice were treated with increasing morin doses (0–5 mg/kg of body weight) for 10 days. Then, (a) novel object recognition test and (b) context novel object recognition tests were performed. After this, the responsive morin concentration of 1 mg/kg was tested for memory tests. (c) Acquisition training started after 6 days of treatment, and (d) retention test was 1 day after treatment with morin. Bars represent the mean ± standard error of the mean (SEM) of 10 animals/group. *p < .05 compared 1 mg/kg of morin‐treated mice versus vehicle‐treated mice, using one‐way ANOVA, repeated measures ANOVA followed by the Tukey post hoc test (Parts A and B). Parts C, D, and E were analyzed with *t*‐student test.

Morin‐treated mice at 1 mg/kg showed a significant increase in the RI in both the NOR and CNOR tests (Figure 2a,b, respectively), suggesting a memory improvement. Moreover, they exhibited significantly improved memory acquisition in the MWM training, as revealed by the faster time to find the hidden platform on the 2nd and 3rd days of training (Figure 2c). Nevertheless, during the memory test 24 h after the last training day, the experimental group did not show any differences in the time spent in the target zone or the number of crosses (Figure 2d).

### 3.2 Morin treatment increased pro‐BDNF, BDNF, and PSD‐95 in the hippocampus and cortex differentially

To determine the possible molecular mechanism involved in memory improvement in mice treated with morin, we evaluated the expression of PSD‐95, an abundant postsynaptic protein of excitatory synapsis that plays a key role in regulating synaptic strength (Keith & El‐Husseini, 2008). In addition, we analyzed pro‐BDNF and BDNF, a neuronal growth factor well known for its involvement in memory processes.

**FIGURE 3:**
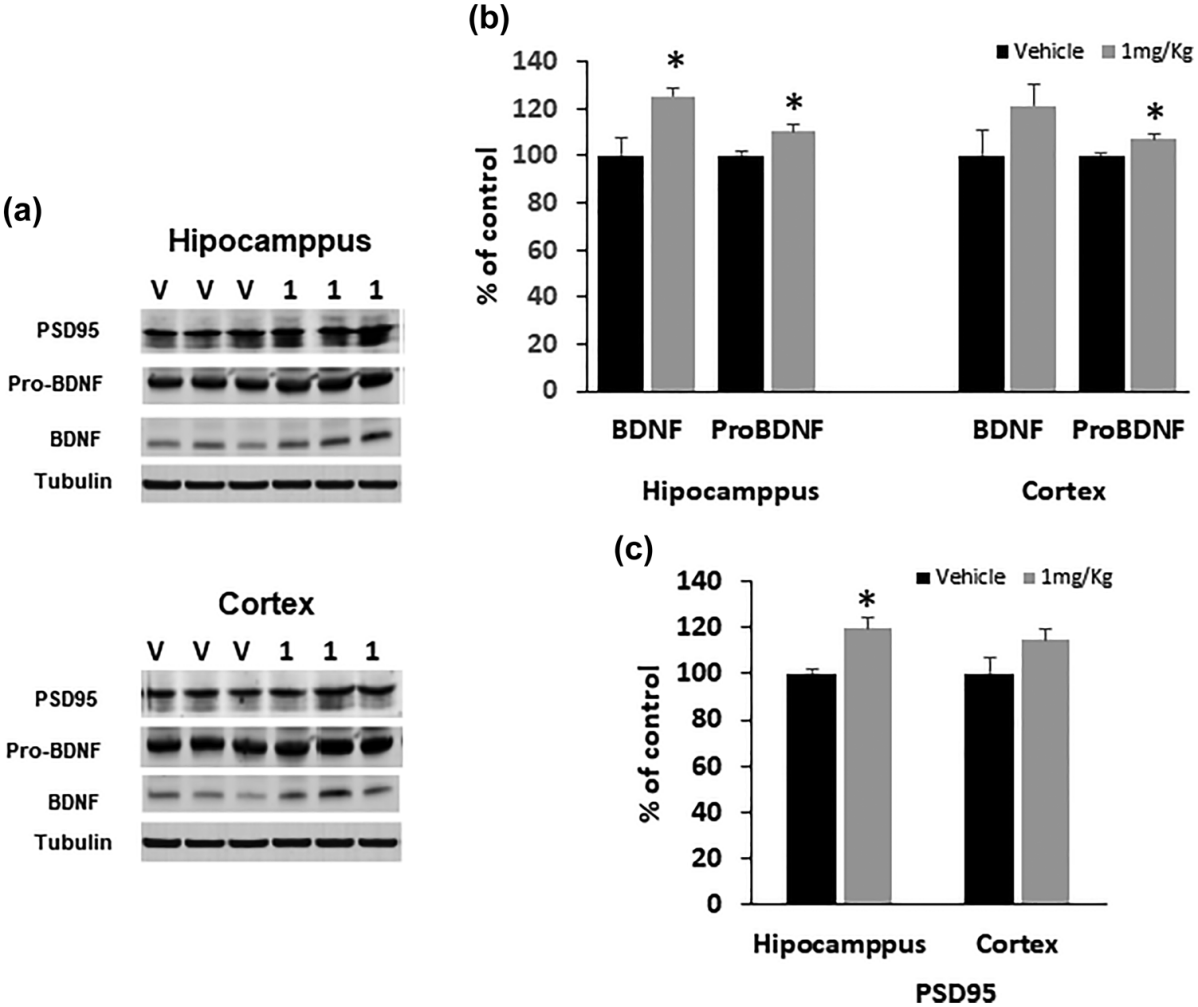
Morin effect on neuronal growth factors and synaptic proteins. Fresh brain tissue was extracted from C57BL/6 mice treated with 1 mg/kg morin for 10 days. (a) Representative immunoblotting and densitometric analysis of the levels of (b) pro‐brain‐derived neurotrophic factor (BDNF) and BDNF and (c) postsynaptic density protein (PSD95) in the hippocampus and cortex are shown. Bars represent the mean ± standard error of the mean (SEM) of three animals/group. *p < .05 compared morin‐treated mice versus vehicle, using *t*‐student test.

These molecular targets were analyzed after the administration of morin at 1 mg/kg in accordance with the memory tests. The results showed that morin significantly increased PSD95 levels in the mouse hippocampus but not in the cortex (Figure 3a,c). Regarding the precursors of BDNF, the pro‐BDNF and the BDNF, morin significantly increased pro‐BDNF in both structures, cortex and hippocampus, but BDNF expression only considerably increased in the hippocampus (Figure 3a,b).

### 3.3 IL‐4 expression does not increase in morin treatment mice

**FIGURE 4:**
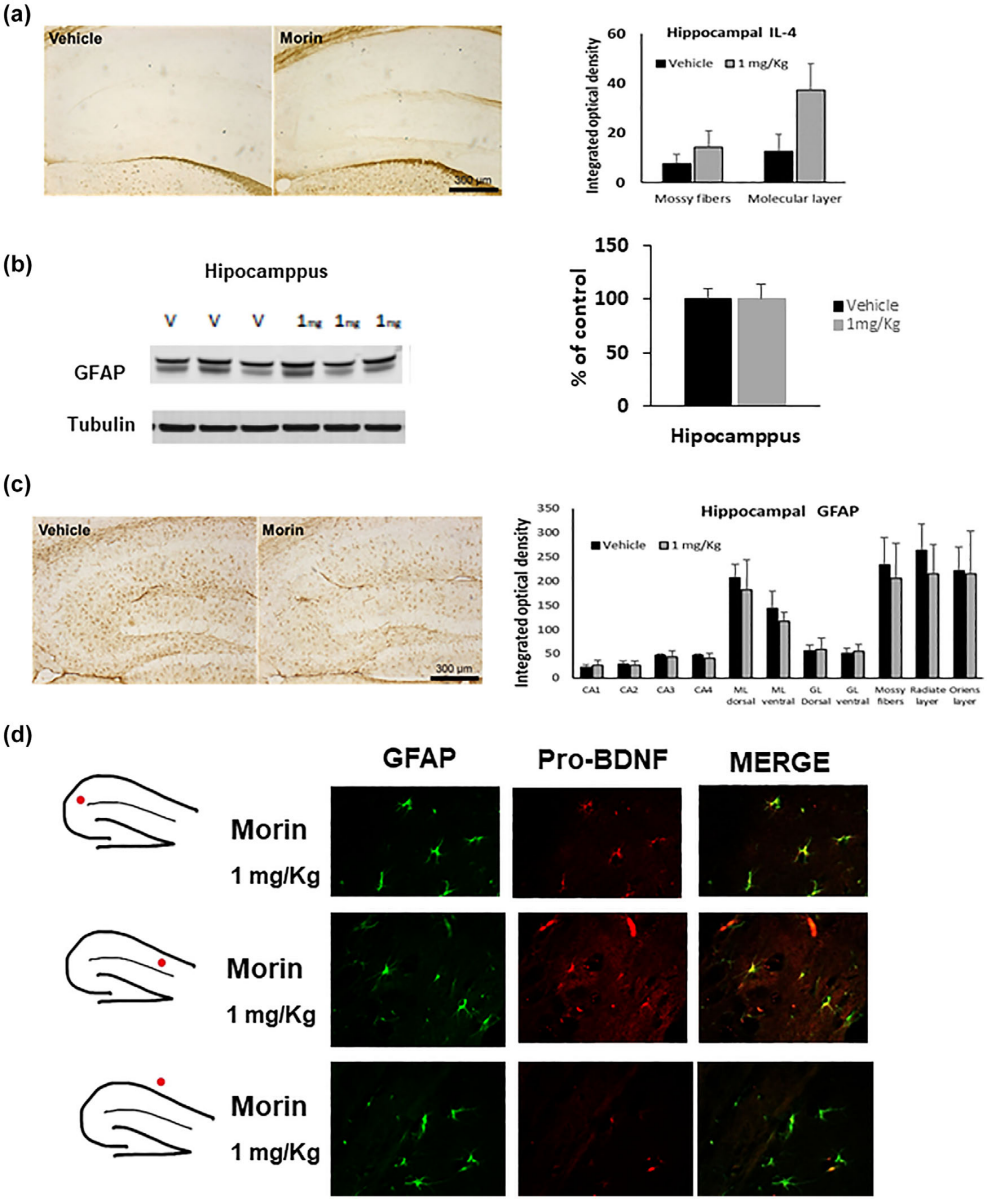
Morin effect on astrocytes. Brain tissue was isolated from C57BL/6 mice treated with 1 mg/kg morin for 10 days. (a) IL‐4 level was determined by immunohistochemistry. GFAP level was also determined by (b) Western blot and (c) immunohistochemistry in the hippocampus. Bars represent the mean ± standard error of the mean (SEM) of three animals/group and compared morin‐treated mice versus vehicle, using *t*‐student test or by one‐way ANOVA. No significant difference was found. Finally, (d) GFAP colocalization with the pro‐brain‐derived neurotrophic factor (BDNF) was performed with immunofluorescence.

One of the molecular mechanisms involved in the increase of BDNF in astrocytes is an extracellular increment of anti‐inflammatory cytokines such as IL‐4 (Gadani et al., 2012); however, we did not find significant changes in the expression of IL‐4 in the hippocampus of mice treated with morin compared to controls (Figure 4a).

### 3.4 GFAP and pro‐BDNF colocalize in the hippocampus in mice treated with morin

Following the same line of research above, we decided to analyze the expression of GFAP in astrocytes. A recent report showed that BDNF induced reactive astrogliosis in the CNS (Ding et al., 2020). Based on this background, we used western blot to analyze the GFAP protein, and we observed no alterations in the morin‐administrated group (Figure 4b). When we analyzed the different hippocampus layers, no significant differences were observed in the number of GFAP‐positive cells compared to the control, despite a decrease in some layers (Figure 4c). Thus, our results show that BDNF does not increase GFAP expression in morin treatment mice. However, another parameter we analyzed to test if astrocytes were involved in the BDNF increase mediated by morin in the hippocampus was the colocalization of GFAP with the BDNF precursor, the pro‐BDNF. Figure 4d shows that GFAP and pro‐BDNF colocalize in the hippocampus in the animals treated with 1 mg/kg of morin, suggesting that the astrocytes could be the source of BDNF in mice stimulated by morin.

### 3.5 Morin treatment increases dendritic complexity

**FIGURE 5:**
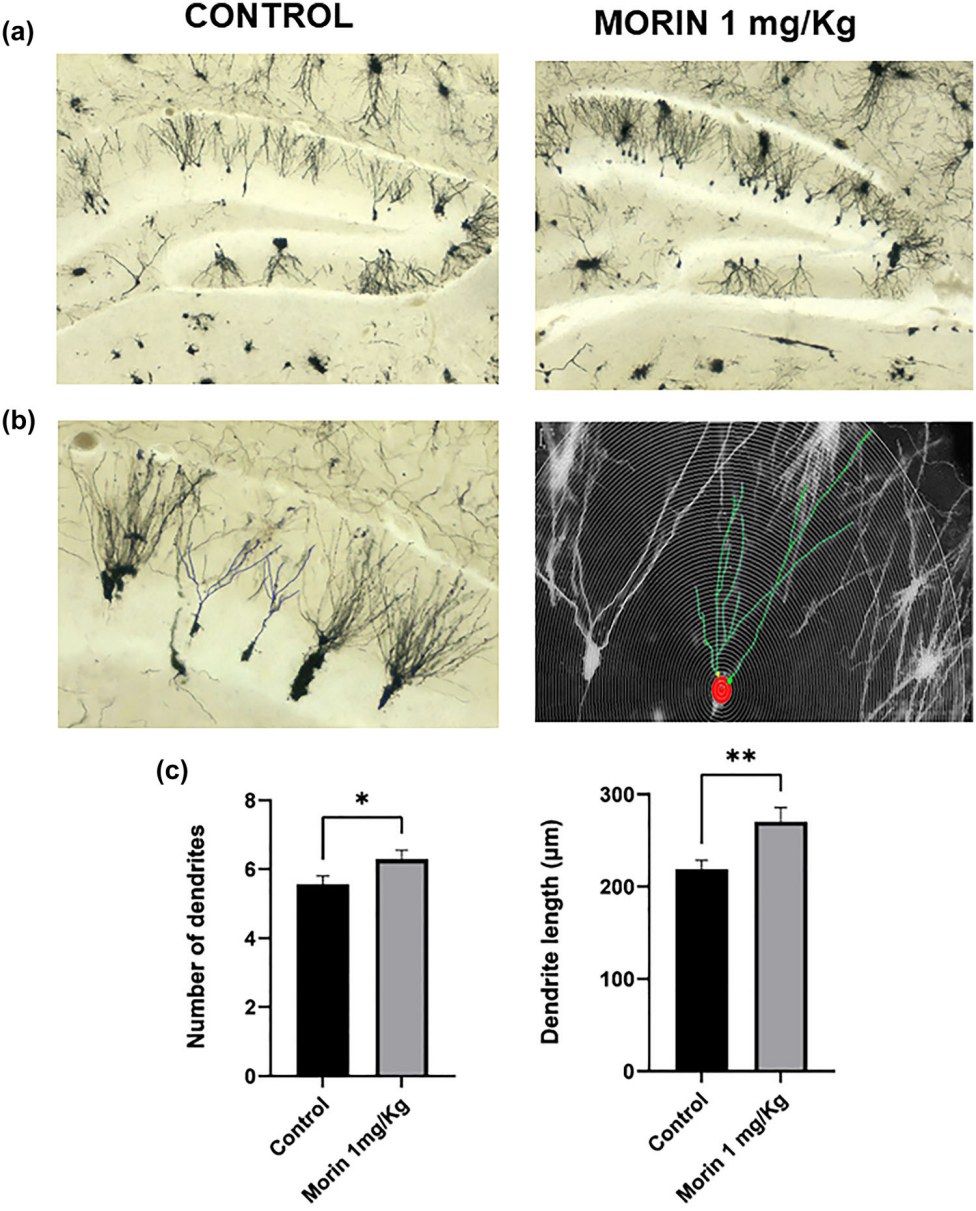
Morin improves synaptic plasticity. Coronal sections of mouse brain from 14‐week old control and treated with 1 mg/kg morin were stained using the Golgi–Cox staining of neural dendritic silver impregnation technique: (a) representative photographs; (b) representative images of Golgi‐positive cells used form the dendritic length and Sholl analysis; (c) graph values of the quantification and extension of the dendrites. Bars represent the mean ± standard error of the mean (SEM) of three animals/group. *p < .05, compare morin‐treated mice versus vehicle, using two‐way student *t*. **p < .001 compared morin‐treated mice versus vehicle, using Mann–Whitney *U* test.

The rise in BDNF is related to the increase in the number and extension of dendrites, which in turn is a reflection of synaptic plasticity and is related to the acquisition of new information (Holtmaat & Caroni, 2016; Zagrebelsky et al., 2020). Our results show that 1 mg/kg morin increases the number and length of neuronal dendrites compared to the control. In addition, morin increases the extension of dendrites (Figure 5a,b). All the results shown here explain in part how morin improves mouse memory.

## 4 DISCUSSION

Strong evidence suggests that dietary flavonoid intake can improve cognitive function in healthy people (Devore et al., 2012; Godos et al., 2020; Travica et al., 2020), delaying the impairment of age‐related cognitive function. Animal studies using diets containing flavonoids have shown that these compounds can improve learning and memory function in healthy animals (Joseph et al., 1998; Shif et al., 2006; Vauzour et al., 2021; Winter, 1998). The beneficial effects of morin have been demonstrated in numerous in vitro and in vivo studies and include its anti‐oxidant, anti‐inflammatory, antiapoptotic, anticancer, neuroprotective, and neuroplastic properties in different human disease models (Rajput et al., 2021); however, less is known about the effects of pure flavonoids on cognitive functions, providing a causation to these improvements. In the present study, we have shown, for the first time, that morin treatment improves recognition and spatial memory function in healthy animals and increases the levels of pro‐BDNF, BDNF, and PSD95 in the hippocampus cortex and pro‐BDNF in the cortex. Recognition and spatial memory probably involve cortical and subcortical structures typically associated with the perirhinal cortex and hippocampal formation. Solid evidence is that increased BDNF in those structures is associated with memory performance (Falkenberg et al., 1992; Hall et al., 2000; Hopkins et al., 2011; Schaaf et al., 2001). BDNF is a ligand for the TrkB receptor, and their interaction results in the PSD95–TrkB complex, which induces the transport of PSD95 to dendrites and synapses (Ji et al., 2005; Yoshii & Constantine‐Paton, 2007). Furthermore, changes in the number and morphology of dendrites observed in the hippocampus of treated animals are consistent with previous work in which similar results are observed in the neuronal ear (He et al., 2017), and also these changes support memory improvement, as these kinds of modifications are associated with cognitive processes (Zagrebelsky et al., 2020). Moreover, increases in postsynaptic protein PSD95 are related to improvements in different forms of memories, including spatial memory (Fitzgerald et al., 2015). The results also showed an increase in pro‐BDNF in both structures. This molecule is not only the precursor form of BDNF but also participates in another relevant process of plasticity as crucial as LTD, through which there is a refinement of connections by eliminating those abnormalities that are ineffective for the formation of synaptic plasticity, memory, and cognition (Borodinova & Salozhin, 2017; Kowiański et al., 2018). Our study presents evidence that morin can induce improvements in memory but some relevant aspects must be resolved. For example, there is little information available about the pharmacokinetics of morin, and the data that is available suggest that this flavonol has poor bioavailability after oral ingestion (Li et al., 2019; Zhang et al., 2011). In this study, we administrate morin intraperitoneally, which generally shows higher bioavailability than oral administration for small molecules (MW < 5000) like morin (Al Shoyaib et al., 2019); moreover, many studies have consistently demonstrated that morin is able to modify various pathological cascades in different neuropathologies such as psychosocial stress, sleep deprivation, schizophrenia, and Alzheimer's disease resulting in cognitive enhancement including memory and learning, (Ben‐Azu et al., 2019; Ben‐Azu et al., 2018; Ben‐Azu et al., 2020; Du et al., 2016; Elizabeth et al., 2020; Olonode et al., 2019) suggesting that morin and/or its metabolites or conjugated forms can not only cross the blood–brain barrier but also interact with different types of neurotransmitter receptors, in particular serotonin and acetylcholine, which are related to memory‐enhancing effects (Ben‐Azu et al., 2021; Thilakarathna & Rupasinghe, 2013). However, it is necessary to perform more research to confirm this pharmacologic characteristic. This work shows that GFAP colocalizes with pro‐BDNF, suggesting that at least astrocytes are involved in the BDNF secreted stimulated by morin. Still, this fact does not discard that morin could stimulate BDNF synthesis and its secretion from another cell type, such as neurons. Lastly, one gap in this study is that only males were included, due mainly to the idea that behavioral responses may be highly variable due to hormonal changes that occur in the estrous cycle; however, a recent study in healthy mice of both sexes shows that there is no greater variability in females than in males in any parameter evaluated and therefore there is no significant difference in the tests of spatial memory, recognition, or BDNF production (Melgar‐Locatelli et al., 2024). This new information encourages future studies to include both sexes.

## 5 CONCLUSIONS

This study shows that morin improves recognition and spatial memory in healthy adult mice. We suggest that the molecular mechanism for these effects involves the increased production and secretion of BDNF by astrocytes.

## AUTHOR CONTRIBUTIONS

**Hilda Martínez‐Coria**: Conceptualization; investigation; methodology; formal analysis; writing—original draft; resources; writing—review and editing. **Norma Serrano‐García**: Investigation; methodology. **Héctor E. López‐Valdés**: Writing—original draft; writing—review and editing; data curation; formal analysis; validation; software. **Gabriela Sinaí López‐Chávez**: Investigation; methodology. **José Rivera‐Alvarez**: Investigation; methodology. **Ángeles Romero‐Hernández**: Investigation; methodology. **Francisca Fernández Valverde**: Investigation; methodology. **Marisol Orozco‐Ibarra**: Investigation; writing—review and editing; data curation; software. **Mónica Adriana Torres‐Ramos**: Conceptualization; investigation; methodology; funding acquisition; writing—review and editing; formal analysis; supervision; project administration; resources; validation; visualization.

## CONFLICT OF INTEREST STATEMENT

All other authors have no conflicts of interest. We declare that we do not have any commercial or associative interest that represents a conflict of interest in connection with the work submitted.

### PEER REVIEW

The peer review history for this article is available at https://publons.com/publon/10.1002/brb3.3444.

## Data Availability

The data that support the findings of this study are available from the corresponding author upon reasonable request.

## References

[brb33444-bib-0001] Al Shoyaib, A. , Archie, S. R. , & Karamyan, V. T. (2019). Intraperitoneal route of drug administration: Should it be used in experimental animal studies? Pharmaceutical Research, 37, 12. 10.1007/s11095-019-2745-x 31873819 PMC7412579

[brb33444-bib-0002] Antunes, M. , & Biala, G. (2012). The novel object recognition memory: Neurobiology, test procedure, and its modifications. Cognitive Processing, 13, 93–110. 10.1007/s10339-011-0430-z 22160349 PMC3332351

[brb33444-bib-0003] Basile, A. , Sorbo, S. , Giordano, S. , Ricciardi, L. , Ferrara, S. , Montesano, D. , Castaldo Cobianchi, R. , Vuotto, M. L. , & Ferrara, L. (2000). Antibacterial and allelopathic activity of extract from *Castanea sativa* leaves. Fitoterapia, 71, (Suppl 1), S110–S116. 10.1016/S0367-326X(00)00185-4 10930721

[brb33444-bib-0004] Bekinschtein, P. , Cammarota, M. , Izquierdo, I. , & Medina, J. H. (2008). BDNF and memory formation and storage. Neuroscientist, 14, 147–156. 17911219 10.1177/1073858407305850

[brb33444-bib-0005] Ben‐Azu, B. , Aderibigbe, A. O. , Ajayi, A. M. , Eneni, A.‐E. O. , Omogbiya, I. A. , Owoeye, O. , Umukoro, S. , & Iwalewa, E. O. (2019). Morin decreases cortical pyramidal neuron degeneration via inhibition of neuroinflammation in mouse model of schizophrenia. International Immunopharmacology, 70, 338–353. 10.1016/j.intimp.2019.02.052 30852289

[brb33444-bib-0006] Ben‐Azu, B. , Aderibigbe, A. O. , Ajayi, A. M. , Eneni, A.‐E. O. , Umukoro, S. , & Iwalewa, E. O. (2018). Involvement of GABAergic, BDNF and Nox‐2 mechanisms in the prevention and reversal of ketamine‐induced schizophrenia‐like behavior by morin in mice. Brain Research Bulletin, 139, 292–306. 10.1016/j.brainresbull.2018.03.006 29548911

[brb33444-bib-0007] Ben‐Azu, B. , Aderibigbe, A. O. , Ajayi, A. M. , Omogbiya, I. A. , Uruaka, C. I. , Umukoro, S. , & Iwalewa, E. O. (2021). Evaluation of the role of monoaminergic and nonmonoaminergic systems in the psychotropic effects of morin in mice: An interaction study with receptor blockers. Nutrire, 46, 8. 10.1186/s41110-021-00137-5

[brb33444-bib-0008] Ben‐Azu, B. , Aderibigbe, A. O. , Eneni, A.‐E. O. , Ajayi, A. M. , Umukoro, S. , & Iwalewa, E. O. (2018). Morin attenuates neurochemical changes and increased oxidative/nitrergic stress in brains of mice exposed to ketamine: Prevention and reversal of schizophrenia‐like symptoms. Neurochemical Research, 43, 1745–1755. 10.1007/s11064-018-2590-z 29956036

[brb33444-bib-0009] Ben‐Azu, B. , Aderibigbe, A. O. , Omogbiya, I. A. , Ajayi, A. M. , & Iwalewa, E. O. (2018). Morin pretreatment attenuates schizophrenia‐like behaviors in experimental animal models. Drug Research, 68, 159–167. 28962045 10.1055/s-0043-119127

[brb33444-bib-0010] Ben‐Azu, B. , Aderibigbe, A. O. , Omogbiya, I. A. , Ajayi, A. M. , Owoeye, O. , Olonode, E. T. , & Iwalewa, E. O. (2018). Probable mechanisms involved in the antipsychotic‐like activity of morin in mice. Biomedecine & Pharmacother, 105, 1079–1090. 10.1016/j.biopha.2018.06.057 30021344

[brb33444-bib-0011] Ben‐Azu, B. , Emokpae, O. , Ajayi, A. M. , Jarikre, T. A. , Orhode, V. , Aderibigbe, A. O. , Umukoro, S. , & Iwalewa, E. O. (2020). Repeated psychosocial stress causes glutamic acid decarboxylase isoform‐67, oxidative‐Nox‐2 changes and neuroinflammation in mice: Prevention by treatment with a neuroactive flavonoid, morin. Brain Research, 1744, 146917. 10.1016/j.brainres.2020.146917 32474018

[brb33444-bib-0012] Borodinova, A. A. , & Salozhin, S. V. (2017). Differences in the biological functions of BDNF and proBDNF in the central nervous system. Neuroscience and Behavioral Physiology, 47, 251–265. 10.1007/s11055-017-0391-5

[brb33444-bib-0013] Brigadski, T. , & Leßmann, V. (2020). The physiology of regulated BDNF release. Cell and Tissue Research, 382, 15–45. 10.1007/s00441-020-03253-2 32944867 PMC7529619

[brb33444-bib-0014] Caruana, M. , Neuner, J. , Högen, T. , Schmidt, F. , Kamp, F. , Scerri, C. , Giese, A. , & Vassallo, N. (2012). Polyphenolic compounds are novel protective agents against lipid membrane damage by α‐synuclein aggregates in vitro. Biochimica Et Biophysica Acta, 1818, 2502–2510. 10.1016/j.bbamem.2012.05.019 22634381

[brb33444-bib-0015] Caselli, A. , Cirri, P. , Santi, A. , & Paoli, P. (2016). Morin: A promising natural drug. Current Medicinal Chemistry, 23, 774–791. 10.2174/0929867323666160106150821 26018232

[brb33444-bib-0016] Chen, P. , Ban, W. , Wang, W. , You, Y. , & Yang, Z. (2023). The devastating effects of sleep deprivation on memory: Lessons from rodent models. Clocks & Sleep, 5, 276–294. 10.3390/clockssleep5020022 37218868 PMC10204456

[brb33444-bib-0017] de Abreu Costa, L. , Henrique Fernandes Ottoni, M. , Dos Santos, M. G. , Meireles, A. B. , Gomes de Almeida, V. , de Fátima Pereira, W. , Alves de Avelar‐Freitas, B. , & Eustáquio Alvim Brito‐Melo, G. (2017). Dimethyl sulfoxide (DMSO) decreases cell proliferation and TNF‐α, IFN‐γ, and IL‐2 cytokines production in cultures of peripheral blood lymphocytes. Molecules (Basel, Switzerland), 22, 1789. 29125561 10.3390/molecules22111789 PMC6150313

[brb33444-bib-0018] Devore, E. E. , Kang, J. H. , Breteler, M. M. B. , & Grodstein, F. (2012). Dietary intakes of berries and flavonoids in relation to cognitive decline. Annals of Neurology, 72, 135–143. 10.1002/ana.23594 22535616 PMC3582325

[brb33444-bib-0019] Ding, H. , Chen, J. , Su, M. , Lin, Z. , Zhan, H. , Yang, F. , Li, W. , Xie, J. , Huang, Y. , Liu, X. , Liu, B. , & Zhou, X. (2020). BDNF promotes activation of astrocytes and microglia contributing to neuroinflammation and mechanical allodynia in cyclophosphamide‐induced cystitis. Journal of Neuroinflammation, 17, 19. 10.1186/s12974-020-1704-0 31931832 PMC6958761

[brb33444-bib-0020] Du, Y. , Qu, J. , Zhang, W. , Bai, M. , Zhou, Q. , Zhang, Z. , Li, Z. , & Miao, J. (2016). Morin reverses neuropathological and cognitive impairments in APPswe/PS1dE9 mice by targeting multiple pathogenic mechanisms. Neuropharmacology, 108, 1–13. 10.1016/j.neuropharm.2016.04.008 27067919

[brb33444-bib-0021] Ebrahimi, A. , & Schluesener, H. (2012). Natural polyphenols against neurodegenerative disorders: Potentials and pitfalls. Ageing Research Reviews, 11, 329–345. 10.1016/j.arr.2012.01.006 22336470

[brb33444-bib-0022] Elizabeth, A. , Adegbuyi, A. , Olusegun, A. , Benneth, B.‐A. , Anthony, E. , Abayomi, A. , & Solomon, U. (2020). Morin hydrate attenuates chronic stress‐induced memory impairment and degeneration of hippocampal subfields in mice: The role of oxidative, nitrergic and neuroinflammatory pathways. Metabolic Brain Disease, 35, 1145–1156. 10.1007/s11011-020-00595-2 32653975

[brb33444-bib-0023] Falkenberg, T. , Mohammed, A. K. , Henriksson, B. , Persson, H. , Winblad, B. , & Lindefors, N. (1992). Increased expression of brain‐derived neurotrophic factor mRNA in rat hippocampus is associated with improved spatial memory and enriched environment. Neuroscience Letters, 138, 153–156. 10.1016/0304-3940(92)90494-R 1407655

[brb33444-bib-0024] Fitzgerald, P. J. , Pinard, C. R. , Camp, M. C. , Feyder, M. , Sah, A. , Bergstrom, H. C. , Graybeal, C. , Liu, Y. , Schlüter, O. M. , Grant, S. G. , Singewald, N. , Xu, W. , & Holmes, A. (2015). Durable fear memories require PSD‐95. Molecular Psychiatry, 20, 901–912. 10.1038/mp.2014.161 25510511 PMC4469631

[brb33444-bib-0025] Gadani, S. P. , Cronk, J. C. , Norris, G. T. , & Kipnis, J. (2012). IL‐4 in the brain: A cytokine to remember. The Journal of Immunology, 189, 4213–4219. 10.4049/jimmunol.1202246 23087426 PMC3481177

[brb33444-bib-0026] Godos, J. , Caraci, F. , Castellano, S. , Currenti, W. , Galvano, F. , Ferri, R. , & Grosso, G. (2020). Association between dietary flavonoids intake and cognitive function in an Italian cohort. Biomolecules, 10, 1300. 10.3390/biom10091300 32916935 PMC7565262

[brb33444-bib-0027] Gong, E. J. , Park, H. R. , Kim, M. E. , Piao, S. , Lee, E. , Jo, D.‐G. , Chung, H. Y. , Ha, N.‐C. , Mattson, M. P. , & Lee, J. (2011). Morin attenuates tau hyperphosphorylation by inhibiting GSK3β. Neurobiology of Disease, 44, 223–230. 10.1016/j.nbd.2011.07.005 21782947 PMC3166962

[brb33444-bib-0028] Gottlieb, M. , Leal‐Campanario, R. , Campos‐Esparza, M. R. , Sánchez‐Gómez, M. V. , Alberdi, E. , Arranz, A. , Delgado‐García, J. M. , Gruart, A. , & Matute, C. (2006). Neuroprotection by two polyphenols following excitotoxicity and experimental ischemia. Neurobiology of Disease, 23, 374–386. 10.1016/j.nbd.2006.03.017 16806951

[brb33444-bib-0029] Hall, J. , Thomas, K. L. , & Everitt, B. J. (2000). Rapid and selective induction of BDNF expression in the hippocampus during contextual learning. Nature Neuroscience, 3, 533–535. 10.1038/75698 10816306

[brb33444-bib-0030] He, Q. , Jia, Z. , Zhang, Y. , & Ren, X. (2017). Morin hydrate promotes inner ear neural stem cell survival and differentiation and protects cochlea against neuronal hearing loss. Journal of Cellular and Molecular Medicine, 21, 600–608. 10.1111/jcmm.13005 27709784 PMC5323642

[brb33444-bib-0031] Holtmaat, A. , & Caroni, P. (2016). Functional and structural underpinnings of neuronal assembly formation in learning. Nature Neuroscience, 19, 1553–1562. 10.1038/nn.4418 27749830

[brb33444-bib-0032] Hopkins, M. E. , Nitecki, R. , & Bucci, D. J. (2011). Physical exercise during adolescence versus adulthood: Differential effects on object recognition memory and brain‐derived neurotrophic factor levels. Neuroscience, 194, 84–94. 10.1016/j.neuroscience.2011.07.071 21839807 PMC3183160

[brb33444-bib-0033] Ibarretxe, G. , Sánchez‐Gómez, M. V. , Campos‐Esparza, M. R. , Alberdi, E. , & Matute, C. (2006). Differential oxidative stress in oligodendrocytes and neurons after excitotoxic insults and protection by natural polyphenols. Glia, 53, 201–211. 10.1002/glia.20267 16206167

[brb33444-bib-0034] Ji, Y. , Pang, P. T. , Feng, L. , & Lu, B. (2005). Cyclic AMP controls BDNF‐induced TrkB phosphorylation and dendritic spine formation in mature hippocampal neurons. Nature Neuroscience, 8, 164–172. 10.1038/nn1381 15665879

[brb33444-bib-0035] Joseph, J. A. , Shukitt‐Hale, B. , Denisova, N. A. , Prior, R. L. , Cao, G. , Martin, A. , Taglialatela, G. , & Bickford, P. C. (1998). Long‐term dietary strawberry, spinach, or vitamin E supplementation retards the onset of age‐related neuronal signal‐transduction and cognitive behavioral deficits. The Journal of Neuroscience, 18, 8047–8055. 10.1523/JNEUROSCI.18-19-08047.1998 9742171 PMC6792999

[brb33444-bib-0036] Keith, D. (2008). Excitation control: Balancing PSD‐95 function at the synapse. Frontiers in Molecular Neuroscience, 1, 4. 10.3389/neuro.02.004.2008 18946537 PMC2526002

[brb33444-bib-0037] Kim, H. , Park, B.‐S. , Lee, K.‐G. , Choi, C. Y. , Jang, S. S. , Kim, Y.‐H. , & Lee, S.‐E. (2005). Effects of naturally occurring compounds on fibril formation and oxidative stress of beta‐amyloid. Journal of Agricultural and Food Chemistry, 53, 8537–8541. 10.1021/jf051985c 16248550

[brb33444-bib-0038] Kowiański, P. , Lietzau, G. , Czuba, E. , Waśkow, M. , Steliga, A. , & Moryś, J. (2018). BDNF: A key factor with multipotent impact on brain signaling and synaptic plasticity. Cellular and Molecular Neurobiology, 38, 579–593. 10.1007/s10571-017-0510-4 28623429 PMC5835061

[brb33444-bib-0039] Lee, J. Y. , Park, H. R. , Moon, S. O. , Kwon, Y. J. , & Rhee, S. J. (2004). Identification and quantification of anthocyanins and flavonoid in mulberry (*Morus* sp.) cultivars. Food Science and Biotechnology, 13(2), 176–184.

[brb33444-bib-0040] Lemkul, J. A. , & Bevan, D. R. (2010). Destabilizing Alzheimer's Abeta(42) protofibrils with morin: Mechanistic insights from molecular dynamics simulations. Biochemistry, 49, 3935–3946. 10.1021/bi1000855 20369844

[brb33444-bib-0041] Li, J. , Yang, Y. , Ning, E. , Peng, Y. , & Zhang, J. (2019). Mechanisms of poor oral bioavailability of flavonoid Morin in rats: From physicochemical to biopharmaceutical evaluations. European Journal of Pharmaceutical Sciences, 128, 290–298. 30557605 10.1016/j.ejps.2018.12.011

[brb33444-bib-0042] Lu, B. , Nagappan, G. , & Lu, Y. (2014). BDNF and synaptic plasticity, cognitive function, and dysfunction. The Handbook of Experimental Pharmacology, 220, 223–250. 10.1007/978-3-642-45106-5_9 24668475

[brb33444-bib-0043] Martinez‐Coria, H. , Green, K. N. , Billings, L. M. , Kitazawa, M. , Albrecht, M. , Rammes, G. , Parsons, C. G. , Gupta, S. , Banerjee, P. , & Laferla, F. M. (2010). Memantine improves cognition and reduces Alzheimer's‐like neuropathology in transgenic mice. American Journal of Pathology, 176, 870–880. 10.2353/ajpath.2010.090452 20042680 PMC2808092

[brb33444-bib-0044] Melgar‐Locatelli, S. , Mañas‐Padilla, M. C. , Gavito, A. L. , Rivera, P. , Rodríguez‐Pérez, C. , Castilla‐Ortega, E. , & Castro‐Zavala, A. (2024). Sex‐specific variations in spatial reference memory acquisition: Insights from a comprehensive behavioral test battery in C57BL/6JRj mice. Behavioural Brain Research, 459, 114806. 10.1016/j.bbr.2023.114806 38086456

[brb33444-bib-0045] Miranda, M. , Morici, J. F. , Zanoni, M. B. , & Bekinschtein, P. (2019). Brain‐derived neurotrophic factor: A key molecule for memory in the healthy and the pathological brain. Frontiers in Cellular Neuroscience, 13, 363. 10.3389/fncel.2019.00363 31440144 PMC6692714

[brb33444-bib-0046] Mizuno, M. , Yamada, K. , Takei, N. , Tran, M. H. , He, J. , Nakajima, A. , Nawa, H. , & Nabeshima, T. (2003). Phosphatidylinositol 3‐kinase: A molecule mediating BDNF‐dependent spatial memory formation. Molecular Psychiatry, 8, 217–224. 10.1038/sj.mp.4001215 12610654

[brb33444-bib-0047] Murman, D. L. (2015). The impact of age on cognition. Seminars in Hearing, 36, 111–121. 10.1055/s-0035-1555115 27516712 PMC4906299

[brb33444-bib-0048] Nakanishi, I. , Ohkubo, K. , Shoji, Y. , Fujitaka, Y. , Shimoda, K. , Matsumoto, K.‐I. , Fukuhara, K. , & Hamada, H. (2020). Relationship between the radical‐scavenging activity of selected flavonols and thermodynamic parameters calculated by density functional theory. Free Radical Research, 54, 535–539. 10.1080/10715762.2020.1813887 32838569

[brb33444-bib-0049] Noor, H. , Cao, P. , & Raleigh, D. P. (2012). Morin hydrate inhibits amyloid formation by islet amyloid polypeptide and disaggregates amyloid fibers. Protein Science, 21, 373–382. 10.1002/pro.2023 22238175 PMC3375438

[brb33444-bib-0050] Olonode, E. T. , Aderibigbe, A. O. , Adeoluwa, O. A. , Eduviere, A. T. , & Ben‐Azu, B. (2019). Morin hydrate mitigates rapid eye movement sleep deprivation‐induced neurobehavioural impairments and loss of viable neurons in the hippocampus of mice. Behavioural Brain Research, 356, 518–525. 10.1016/j.bbr.2017.12.024 29284109

[brb33444-bib-0051] Panche, A. N. , Diwan, A. D. , & Chandra, S. R. (2016). Flavonoids: An overview. Journal of Nutritional Science, 5, e47. 10.1017/jns.2016.41 28620474 PMC5465813

[brb33444-bib-0052] Pandey, K. B. , & Rizvi, S. I. (2009). Plant polyphenols as dietary antioxidants in human health and disease. Oxidative Medicine and Cellular Longevity, 2, 270–278. 10.4161/oxim.2.5.9498 20716914 PMC2835915

[brb33444-bib-0053] Pongsak Rattanachaikunsopon, P. , & Phumkhachorn, P. (2010). Contents and antibacterial activity of flavonoids extracted from leaves of *Psidium guajava* . Journal of Medicinal Plants Research, 4, 393–396.

[brb33444-bib-0054] Poo, M.‐M. (2001). Neurotrophins as synaptic modulators. Nature Reviews Neuroscience, 2, 24–32. 10.1038/35049004 11253356

[brb33444-bib-0055] Rajput, S. A. , Wang, X.‐Q. , & Yan, H.‐C. (2021). Morin hydrate: A comprehensive review on novel natural dietary bioactive compound with versatile biological and pharmacological potential. Biomedecine & Pharmacother, 138, 111511. 10.1016/j.biopha.2021.111511 33744757

[brb33444-bib-0056] Schaaf, M. J. M. , Workel, J. O. , Lesscher, H. M. , Vreugdenhil, E. , Oitzl, M. S. , & Ron De Kloet, E. (2001). Correlation between hippocampal BDNF mRNA expression and memory performance in senescent rats. Brain Research, 915, 227–233. 10.1016/S0006-8993(01)02855-4 11595212

[brb33444-bib-0057] Serrano‐García, N. , Fernández‐Valverde, F. , Luis‐Garcia, E. R. , Granados‐Rojas, L. , Juárez‐Zepeda, T. E. , Orozco‐Suárez, S. A. , Pedraza‐Chaverri, J. , Orozco‐Ibarra, M. , & Jiménez‐Anguiano, A. (2018). Docosahexaenoic acid protection in a rotenone induced Parkinson's model: Prevention of tubulin and synaptophysin loss, but no association with mitochondrial function. Neurochemistry International, 121, 26–37. 10.1016/j.neuint.2018.10.015 30342962

[brb33444-bib-0058] Shif, O. , Gillette, K. , Damkaoutis, C. M. , Carrano, C. , Robbins, S. J. , & Hoffman, J. R. (2006). Effects of *Ginkgo biloba* administered after spatial learning on water maze and radial arm maze performance in young adult rats. Pharmacology Biochemistry and Behavior, 84, 17–25. 10.1016/j.pbb.2006.04.003 16740301

[brb33444-bib-0059] Spencer, J. P. E. (2008). Food for thought: The role of dietary flavonoids in enhancing human memory, learning and neuro‐cognitive performance. Proceedings of the Nutrition Society, 67, 238–252. 10.1017/S0029665108007088 18412998

[brb33444-bib-0060] Stangl, D. , & Thuret, S. (2009). Impact of diet on adult hippocampal neurogenesis. Genes & Nutrition, 4, 271–282. 19685256 10.1007/s12263-009-0134-5 PMC2775886

[brb33444-bib-0061] Thilakarathna, S. , & Rupasinghe, H. (2013). Flavonoid bioavailability and attempts for bioavailability enhancement. Nutrients, 5, 3367–3387. 10.3390/nu5093367 23989753 PMC3798909

[brb33444-bib-0062] Travica, N. , D'cunha, N. M. , Naumovski, N. , Kent, K. , Mellor, D. D. , Firth, J. , Georgousopoulou, E. N. , Dean, O. M. , Loughman, A. , Jacka, F. , & Marx, W. (2020). The effect of blueberry interventions on cognitive performance and mood: A systematic review of randomized controlled trials. Brain, Behavior, and Immunity, 85, 96–105. 10.1016/j.bbi.2019.04.001 30999017

[brb33444-bib-0063] Vauzour, D. , Rendeiro, C. , D'amato, A. , Waffo‐Téguo, P. , Richard, T. , Mérillon, J. M. , Pontifex, M. G. , Connell, E. , Müller, M. , Butler, L. T. , Williams, C. M. , & Spencer, J. P. E. (2021). Anthocyanins promote learning through modulation of synaptic plasticity related proteins in an animal model of ageing. Antioxidants (Basel Switzerland), 10, 1235. 10.3390/antiox10081235 34439483 PMC8388918

[brb33444-bib-0064] Venkataraman, K. (1972). Wood phenolics in the chemotaxonomy of the *Moraceae* . Phytochemistry, 11, 1571–1586. 10.1016/0031-9422(72)85002-7

[brb33444-bib-0065] Verheijen, M. , Lienhard, M. , Schrooders, Y. , Clayton, O. , Nudischer, R. , Boerno, S. , Timmermann, B. , Selevsek, N. , Schlapbach, R. , Gmuender, H. , Gotta, S. , Geraedts, J. , Herwig, R. , Kleinjans, J. , & Caiment, F. (2019). DMSO induces drastic changes in human cellular processes and epigenetic landscape in vitro. Scientific Reports, 9, 4641. 10.1038/s41598-019-40660-0 30874586 PMC6420634

[brb33444-bib-0066] Vorhees, C. V. , & Williams, M. T. (2006). Morris water maze: Procedures for assessing spatial and related forms of learning and memory. Nature Protocols, 1, 848–858. 10.1038/nprot.2006.116 17406317 PMC2895266

[brb33444-bib-0067] Wang, G. , Gilbert, J. , & Man, H.‐Y. (2012). AMPA receptor trafficking in homeostatic synaptic plasticity: Functional molecules and signaling cascades. Neural Plasticity, 2012, 825364. 10.1155/2012/825364 22655210 PMC3359728

[brb33444-bib-0068] Wijeratne, S. S. K. , Abou‐Zaid, M. M. , & Shahidi, F. (2006). Antioxidant polyphenols in almond and its coproducts. Journal of Agricultural and Food Chemistry, 54, 312–318. 10.1021/jf051692j 16417285

[brb33444-bib-0069] Winter, J. C. (1998). The effects of an extract of *Ginkgo biloba*, EGb 761, on cognitive behavior and longevity in the rat. Physiology & Behavior, 63, 425–433. 10.1016/S0031-9384(97)00464-2 9469738

[brb33444-bib-0070] Yamada, K. , & Nabeshima, T. (2004). Interaction of BDNF/TrkB signaling with NMDA receptor in learning and memory. Drug News & Perspectives, 17, 435–438. 10.1358/dnp.2004.17.7.863702 15514702

[brb33444-bib-0071] Yoshii, A. , & Constantine‐Paton, M. (2007). BDNF induces transport of PSD‐95 to dendrites through PI3K‐AKT signaling after NMDA receptor activation. Nature Neuroscience, 10, 702–711. 10.1038/nn1903 17515902

[brb33444-bib-0072] Zagrebelsky, M. , Tacke, C. , & Korte, M. (2020). BDNF signaling during the lifetime of dendritic spines. Cell and Tissue Research, 382, 185–199. 10.1007/s00441-020-03226-5 32537724 PMC7529616

[brb33444-bib-0073] Zhang, J. , Peng, Q. , Shi, S. , Zhang, Q. , Sun, X. , Gong, T. , & Zhang, Z. (2011). Preparation, characterization, and in vivo evaluation of a self‐nanoemulsifying drug delivery system (SNEDDS) loaded with morin‐phospholipid complex. International Journal of Nanomedicine, 6, 3405–3414. 22267925 10.2147/IJN.S25824 PMC3260034

[brb33444-bib-0074] Zhang, Z.‐T. , Cao, X.‐B. , Xiong, N. , Wang, H.‐C. , Huang, J.‐S. , Sun, S.‐G. , & Wang, T. (2010). Morin exerts neuroprotective actions in Parkinson disease models in vitro and in vivo. Acta Pharmacologica Sinica, 31, 900–906. 10.1038/aps.2010.77 20644549 PMC4007813

